# Peripheral T lymphocytes predict the severity and prognosis in patients with HBV-related acute-on-chronic liver failure

**DOI:** 10.1097/MD.0000000000024075

**Published:** 2021-02-05

**Authors:** Feixia Wang, Weiwei Sun, Qian Xiao, Chongfeng Liang, Shulian Jiang, Yanan Lian, Jiangjuan Shao, Shanzhong Tan, Shizhong Zheng

**Affiliations:** a Department of Integrated TCM and Western Medicine, The Affiliated Nanjing Hospital of Nanjing University of Chinese Medicine; b Jiangsu Key Laboratory for Pharmacology and Safety Evaluation of Chinese Materia Medica, School of Pharmacy, Nanjing University of Chinese Medicine, Nanjing, China.

**Keywords:** acute-on-chronic liver failure, hepatitis B, immune system, peripheral T lymphocytes, prognosis

## Abstract

**Background:**

Hepatitis B virus-related acute-on-chronic liver failure (HBV-ACLF) is a life-threatening syndrome with high mortality. Biomarkers are urgently needed to predict the prognosis of HBV-ACLF. Recent evidence suggests a key role for immune system in the pathology of HBV-ACLF. Here, we analyzed the correlation between peripheral blood T lymphocytes and the severity and prognosis in HBV-ACLF patients.

**Method:**

Sixty-six patients with HBV-ACLF received conventional medical treatments for 4 weeks. Twenty-five healthy subjects and 20 HBV patients were enrolled for comparison. We determined white blood cell count, lymphocytes, CD3^+^, CD4^+^ and CD8^+^ T cells, and CD4^+^CD25^+^ Treg cells in the blood of all subjects. Their associations with laboratory parameters before or after treatments were statistically analyzed.

**Result:**

The results showed that compare normal subjects and chronic hepatitis B patients, HBV-ACLF patients had significantly increased white blood count, CD4^+^ T cells and decreased lymphocytes, CD3^+^ T cells, and Treg cells. Correlation analysis showed that white blood cell, lymphocytes, and peripheral T lymphocytes were correlated with prothrombin activity (PTA) and model for end-stage liver disease (MELD) scores. After treatment, white blood cell, lymphocytes, and peripheral T lymphocytes were also correlated with PTA and MELD scores. Additionally, total bilirubin (TBIL), alanine aminotransferase (ALT), international standard ratio (INR), MELD, and white blood cell count were potential prognostic criteria for HBV-ACLF patients.

**Conclusion:**

HBV-ACLF patients had depletion and dysfunction of immune system. Changes of peripheral T lymphocytes were closely related to the pathogenesis and prognosis of disease. Our results may contribute to predict the severity of HBV-ACLF, and provide a prognosis response to improve the treatment of HBV-ACLF.

## 1 Introduction

Currently, chronic hepatitis B (CHB) remains a major health problem worldwide.^[1]^ In certain cases, CHB patients may have an acute exacerbation and evolve to acute-on-chronic liver failure (ACLF), which named hepatitis B virus (HBV)-related ACLF.^[2,3]^ It has been indicated that alcoholic liver cirrhosis represents 50% to 70% of the underlying liver diseases of ACLF in Western countries, whereas hepatitis-associated liver cirrhosis constitutes 10% to 30% of all ACLF cases.^[4]^ In most Asian countries with high prevalence of hepatitis B virus (HBV) infection, CHB is considered to be the major causative factor for ACLF, accounting for about 70% of ACLF cases and for as little as 15% of alcohol-related ACLF.

Causes of liver failure are diversified. In certain Western countries, alcohol is the main cause, while HBV is the main cause of liver failure in China, and more than 90% of proportion has causes of chronic liver failure.^[5]^ ACLF is a complicated syndrome which causes rapid deterioration of liver function, and leads to a variety of complications, and further leads to multiple organ failure, high mortality, acute onset, and poor prognosis.^[6,7]^ Liver transplantation can significantly reduce the mortality rate of patients, but its clinical application is not extensive due to the lack of liver source and its high cost.^[8,9]^ Early and accurate assessment of patients with ACLF can provide the optimal treatment to improve the outcomes in patients. A limited number of retrospective studies have reported a few specific epidemiological characteristics and an unacceptable high short-term mortality for HBV-ACLF.^[2,10,11]^ Although the research on the pathophysiological mechanism of HBV-ACLF has achieved certain results during recent years, there are breakthroughs in diagnosis and treatment. However, modern medicine still has no specific kind of drugs or treatments for the treatment of HBV-ACLF but mainly symptomatic treatment. Our previous studies have found that immune factors played a crucial role in the pathogenesis of liver failure, especially in the late stage of liver failure. T lymphocytes and their subgroups were dysfunctional, and the frequency was significantly decreased. ^[12]^ Increasing studies found that dendritic cells and cytotoxic T cells have a deletion of phenotype and a decline of function in peripheral blood of patients with ACLF.^[13,14]^ These findings suggest that T lymphocytes and their subgroups are related to the development and prognosis of HBV-ACLF. Here, we postulated that a panel of peripheral blood T lymphocytes could provide additional prognostic information and add it to existing risk prediction algorithms established by generally available clinical and biochemical parameters.

## 2 Method

### 2.1 Patients and study design

Ninety-seven consecutive patients with ACLF caused by CHB were enrolled from the Affiliated Nanjing Hospital of Nanjing University of Chinese Medicine (Nanjing, China) between August 2013 and March 2016. Additionally, 25 normal subjects and 20 CHB patients were screened to facilitate comparison with HBV-ACLF patients. The study followed the tenets of the Declaration of Helsinki, and informed written consents were obtained from all patients followed by explanation of the nature and possible consequences of the study. The study protocol was approved by the Medical Ethical Committee of The Affiliated Nanjing Hospital of Nanjing University of Chinese Medicine. Clinical characteristics and prognostic indicators were collected to develop and validate new diagnostic criteria and prognostic score for HBV-ACLF. Diagnosis of ACLF is based on the 2009 APASL-ACLF consensus guidelines.^[15]^ The inclusion criteria were from age 18 to 70 years old, who were weak with gastrointestinal symptoms such as anorexia, vomiting and abdominal distension, serum total bilirubin (TBil)≥171 μmol/L or daily increase ≥17.1 μmol/L, 30% <prothrombin activity (PTA)≤40% (or 1.5 < INR≤1.9). All the patients were hepatitis B surface antigen (HBsAg)-positive for more than 6 months, but no detection of hepatic encephalopathy or other complications, and no evidence on ultrasonography of active hepatocellular carcinoma. Previous treatment with interferon was considered an exclusion criterion. Patients were excluded from the study if they: were <18 years or >70 years of age; had acute severe hepatitis or subacute severe hepatitis; were pregnant or lactating women; were positive anti-HIV; had severe extrahepatic diseases. During hospitalization, in the 97 HBV-ACLF patients enrolled, 66 patients received a 4-week integrative treatment. The detailed treatment protocol for ACLF is described in the online supplementary materials. The study protocol was approved by the Clinical Research Ethics Committee of the Affiliated Nanjing Hospital of Nanjing University of Chinese Medicine. Appropriate approvals were obtained from patients or their legal surrogates before enrollment.

### 2.2 Clinical data collection

The following clinical data were collected: demographic data, admission causes, history of etiology, and complications associated with acute decompensation or severe liver injury, laboratory measurements (e.g., serum albumin, total bilirubin, alanine aminotransferase, prothrombin activity, sodium and creatinine levels), HBV infection biomarkers, and HBV-DNA levels. Additionally, antiviral treatment for HBV (nucleoside analogues, including lamivudine, adefovir, entecavir, telbivudine, and tenofovir, within 6 months prior to hospitalization) was also collected. Integrative treatments were standardized for all included patients as above. Laboratory data, HBV infection biomarkers, and HBV-DNA levels were measured at weeks 0 and 4.

### 2.3 Profiling the lymphocyte and peripheral blood T lymphocytes of enrolled normal subjects, CHB patients, and HBV-ACLF patients

Lymphocytes and peripheral blood T lymphocytes were collected, and normal subjects and CHB patients were used to facilitate the comparison with HBV-ACLF patients. Peripheral blood total T lymphocytes and HBV-specific CD3^+^, CD4^+^, CD8^+^ T, and CD4^+^CD25^+^ Treg cells were detected by flow cytometry.

### 2.4 The detailed treatment protocol for HBV-ACLF patients

The standard internal medications included routine supportive treatment. All enrolled patients received adequate rest and nutritional support therapy, including intravenous high glucose, vitamin, glutathione, adenosylmethionine, or branched-chain amino acids and the use of albumin or plasma for hypoproteinemia. Patients with the HBV pathogen who were HBV-DNA positive immediately received nucleoside analogs (adefovir dipivoxil, entecavir). Patients with endotoxemia were treated with intestinal microflora modulators or lactulose to regulate intestinal flora. Patients with renal failure were treated with intravenous albumin or renal replacement therapy. Treatments with glycyrrhizic acid such as glycyrrhizin, antioxidants, and microcirculation were used for liver protection. Hepatocyte growth therapy was adopted to reduce persistent hepatocyte damage in patients with ACLF. Extracorporeal liver support systems, including albumin dialysis and plasma exchange, were performed as common therapeutic options for ACLF patients if needed.

### 2.5 Efficacy criteria after a 4-week treatment

Determination to the 2014 APASL-ACLF consensus guidelines. This study is divided into effective and ineffective, and the endpoint is judged as the patient's discharge status or death at the hospital. Effective: patients’ symptoms and signs were basically disappeared, and liver function returned to normal; or the symptoms and signs were obviously restored after treatment, and the liver function was obviously improved. Ineffective: patients’ conditions had not improved significantly after treatment, or patients’ conditions were further developed and even vital signs such as organ failure and other unstable vital signs appeared.

### 2.6 MELD score

The levels of serum creatinine, the INR for prothrombin time (40%≤PTA≤50%), and the levels of serum TBIL of each HBV-ACLF patient were recorded. The MELD score was calculated according to the formula as previously reported ^[16]^. MELD score = 3.78 × ln [TBIL (mg dL^−1^)] + 11.2 × ln INR + 9.57 × ln [creatinine (mg dL^−1^)] + 6.43 × (etiology: 0 if cholestatic or alcoholic, 1 otherwise). Unit conversion: TBIL: 1 mg dL^−1^ = 17.1 μmol^−1^; Cr: 1 mg dL^−1^ = 88.4 μmol L^−1^.

### 2.7 Statistics and analysis

The data were expressed as means ± SD or medians with interquartile range. SPSS software V.25 (SPSS, Chicago, IL) and GraphPad Prism 7 (San Diego, CA) was used for statistical analysis, χ^2^ test for categorical variables, Student *t* test and one-way analysis of variance, the nonparametric Mann–Whitney *U* test, and Kruskal–Wallis H test for continuous variables as appropriate. Spearman rank correlation analysis was used to explore the rank correlation between lymphocyte and peripheral blood T lymphocytes with severity of disease in the entire cohort. The magnitude of the associations was assessed by correlation coefficient *r* and *p* values. Correlation coefficient *r* is a positive value, indicating that the variable has a significant positive linear correlation. The continuous variables in the general clinical data and laboratory test indicators were used as test variables, and the 4-week follow-up results were used as state variables for the ROC curve. The area under the receiver operating characteristic curve (AUROC) of the various values of different prognostic scoring systems was compared by *Z* test using Delong method.

## 3 Results

*Characteristics of HBV-ACLF patients.* A total of 97 patients (68 men [70.10%] and 29 women [29.89%]) with HBV-ACLF were enrolled in the present study. The studied patients had a mean age of 40 ± 11 years. The characteristics of patients are presented in our results (see table, Supplemental Table 1, which illustrates the characteristics of patients), among them 84 (86.60%) had 500–2 × 10^6^ IU/mL HBV-DNA levels, and 13 (13.40%) had less 500 IU/mL HBV-DNA levels. Laboratory indicators of measurements showed the levels of white blood count, serum TBIL, and PTA, and serum sodium and creatinine were also indicated. Meanwhile, there were great variations in albumin (Alb) and aspartate aminotransferase (ALT). Thirty-two patients (32.99%) had HBV etiology with a diagnostic history of CHB, and 16 (16.49%) had HBV etiology with cirrhosis. Nevertheless, 49 patients had no paper medical record, and it is unable to determine etiology. The characteristics of HBV patients are presented in Table S2 among them 15 (75%) had more than 500 IU/mL HBV-DNA levels. Laboratory indicators of measurements showed the levels of white blood count, serum TBIL, and PTA, and serum sodium and creatinine were also indicated (see table, Supplemental Table 2, which illustrates the characteristics of patients).

### 3.1 Comparison of white blood cell count, lymphocyte, and peripheral blood T lymphocytes in HBV-ACLF patients with normal subjects and HBV patients

**Figure 1:**
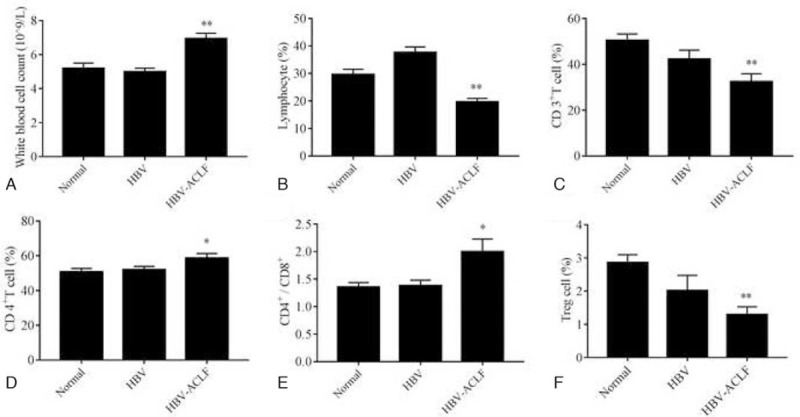
Comparison of white blood cell count and peripheral blood T lymphocytes in HBV-ACLF patients with normal subjects and HBV patients. These histograms indicate white blood cell count and peripheral blood T lymphocytes in HBV-ACLF patients, normal subjects, and HBV patients. A, Levels of white blood cell. B, Proportion of lymphocytes. C, Proportion of CD3^+^T cells. D, Proportion of CD4^+^T cells. E, Ratio of CD4^+^T cells and CD8^+^T cells. F, Proportion of Treg cells. Statistical significance: ^∗^*P* < .05 versus normal subjects, ^∗∗^*P* < .01 versus normal subjects.

We next sought to identify the risk factors predicting the severity of HBV-ACLF patients. We first compared white blood cell count, lymphocytes, and peripheral blood T lymphocytes in HBV-ACLF patients with those in normal subjects and HBV patients. As shown in Table 1 and Figure 1, the advanced HBV-ACLF patients had a significantly increased level (*P* < .05) than normal subjects in white blood cell count, CD4^+^ T cells, and the ratio of CD4^+^ and CD8^+^ T cells (Fig. 1A, D, and E). However, total lymphocytes, CD3^+^ T cells, and Treg cells are significantly decreased (*P* < .01) in HBV-ACLF patients compared with those in normal subjects and CHB patients (Fig. 1B, C, and F). Results suggested that when patients had HBV-ACLF, the frequency of peripheral blood effector T lymphocytes, such as total lymphocytes, CD3^+^ T cells, Treg cells, etc., was inhibited. These implied lymphocytes involved in liver injury-related immune damage were extensively depleted, and it could destroy immune homeostasis. The immune clearance is far greater than the immunosuppression, thus promoting the development of liver failure.

**Table 1:** Comparison of white blood cell count, lymphocyte, and peripheral blood T lymphocytes in HBV-ACLF patients with normal subjects and HBV patients.

Variables	Normal (n = 23)	HBV (n = 23)	HBV-ACLF (n = 97)
WBC (10^9^/L)	5.18 ± 1.59	4.98 ± .96	6.94 ± 3.10^∗∗^
Lymphocyte (%)	29.60 ± 9.37	37.59 ± 9.88	19.68 ± 12.12^∗∗^
CD3^+^ T cell (%)	50.38 ± 13.87	42.21 ± 18.97	32.40 ± 19.09^∗∗^
CD4^+^ T cell (%)	50.75 ± 9.45	51.96 ± 9.25	58.69 ± 14.19^∗^
CD8^+^ T cell (%)	39.17 ± 6.65	40.13 ± 7.98	36.08 ± 12.50
CD4^+^ CD8 +	1.35 ± 0.40	1.38 ± 0.48	1.99 ± 1.26^∗^
Treg cell (%)	2.86 ± 1.16	2.01 ± 2.22	1.30 ± 1.17^∗∗^

### 3.2 Correlation of white blood cell count, lymphocyte, and peripheral blood T lymphocytes with severity of HBV-ACLF disease

**Figure 2:**
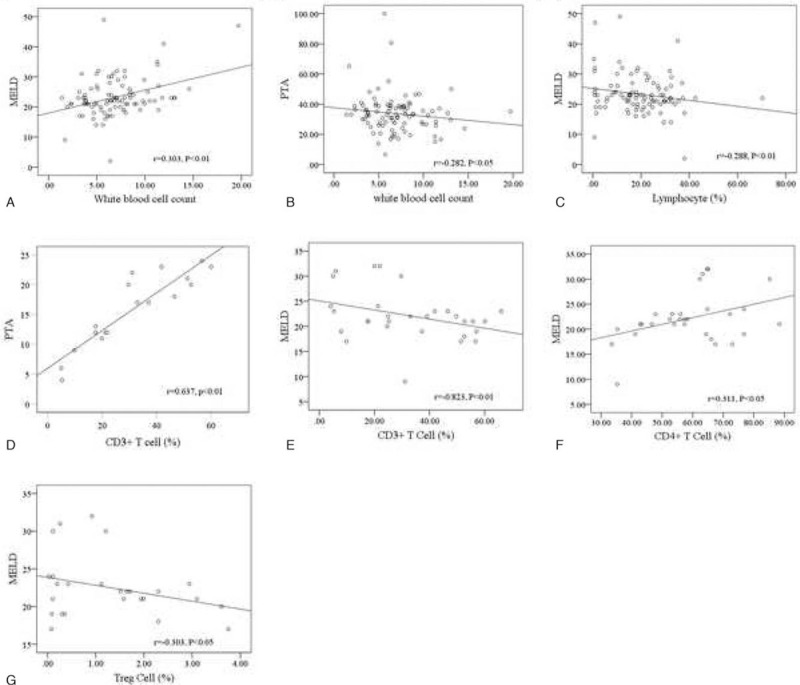
Correlation of white blood cell count, lymphocyte, and peripheral blood T lymphocytes with severity of HBV-ACLF disease. Spearman correlation analysis represents the correlation between indicators. A, White blood cell count is positively correlated with MELD. Statistical significance: *P* < .05. B, White blood cell count is negatively correlated with PTA. Statistical significance: *P* < .05. C, Lymphocytes are negatively correlated with MELD. Statistical significance: *P* < .01. D, CD3^+^T cells are positively correlated with PTA. Statistical significance: *P* < .01. E, CD3^+^T cells are negatively correlated with MELD. Statistical significance: *P* < .01. F, CD4^+^T cells are positively correlated with MELD. Statistical significance: *P* < .05. G, Treg cells are negatively correlated with MELD. Statistical significance: *P* < .05.

PTA reflects the degree of necrosis of hepatocytes in patients with severe hepatitis, and MELD score is calculated from relevant laboratory indicators, which also reflects the severity of disease. There is evidence indicating that the INR and MELD scores are important indicators that may identify patients with a HBV etiology, who have a higher mortality risk.^[17]^ In our current study, Spearman correlation analysis indicated that white blood cell count and CD4^+^ T cells were positively correlated with MELD score, and CD3^+^ T cells were positively correlated with PTA (Fig. 2A D, and F), while MELD score was negatively correlated with lymphocytes, CD3^+^ T cells, CD4^+^CD25^+^ Treg cells and PTA was negatively correlated with white blood cell count (Fig. 2B, C, E, and G). The results indicated that white blood cell count, lymphocyte, and peripheral blood T lymphocytes were obviously correlated with the severity of HBV-ACLF. This might be a hint that changes in the proportion of T lymphocyte subsets could be used as a diagnostic criterion for patients with HBV-ACLF.

### 3.3 Differences of laboratory indicators and peripheral blood T lymphocytes of HBV-ACLF patients after 4-week integrative treatment

**Figure 3:**
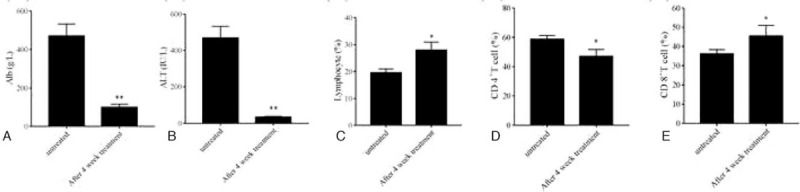
Changes of laboratory indicators and peripheral blood T lymphocytes in HBV-ACLF patients after 4-week integrative treatment. A, Levels of Alb. B, Levels of ALT. C, Proportion of lymphocytes. D, Proportion of CD4^+^T cells. E, Proportion of CD8 + T cells. Statistical significance: ^∗^*P* < .05 versus before treatment, ^∗∗^*P* < .01 versus before treatment.

It was previously demonstrated that entecavir treatment prevented disease progression and increased the survival of patients with HBV-ACLF.^[18]^ In our study, anti-virus drugs, hepatinica, and other hepatocyte growth-promoting factors were used for treatment. Among the 97 patients enrolled, 66 received 4-week treatment. We tested and analyzed the changes and differences of characteristics and peripheral blood T lymphocytes in HBV-ACLF patients after treatment. Table 2 demonstrated the differences of laboratory indicators and peripheral blood T lymphocytes in HBV-ACLF patients after the 4-week integrative treatment. Results indicated that Alb and ALT showed a great variety after treatment (Fig. 3A and B). We also compared the changes of immune system of patients before and after treatment. The rising trend of CD4^+^T cells and the decreasing trend of the frequency of lymphocytes and CD8^+^T cells were intervened after treatment (Fig. 3C–E). The results suggested that liver function and immune function in patients were improved after the 4-week integrative treatment.

**Table 2:** Differences of laboratory indicators and peripheral blood T lymphocytes of HBV-ACLF patients after 4-week integrative treatment.

	HBV-ACLF (n = 66)			
Variables	After 4-week treatment	Difference with untreated	Z value	*P* value
WBC (10^9^/L)	7.20 ± 4.91	1.41 ± 6.25	−1.317	.188
TBIL (μmol/L)	356.86 ± 197.49	−34.60 ± 212.22	−1.017	.309
Alb (g/L)	99.89 ± 136.04	–369.84 ± 615.40	−5.963	.004^∗∗^
ALT (IU/L)	69.87 ± 42.49	–376.97 ± 615.96	−1.040	.005^∗∗^
PTA (%)	36.03 ± 13.07	2.03 ± 20.68	−0.324	.746
Serum sodium (μmol/L)	132.51 ± 7.58	1.91 ± 7.78	−1.935	.051
Creatinine (μmol/L)	75.73 ± 29.66	15.64 ± 77.61	−1.537	.124
Lymphocyte (%)	22.59 ± 15.69	−0.94 ± 16.34	−2.420	.047^∗^
CD3^+^ T cell (%)	24.41 ± 12.80	−0.91 ± 34.08	0.392	.706
CD8^+^ T cell (%)	49.01 ± 8.77	11.07 ± 15.45	−2.416	.011^∗^
CD4^+^ T cell (%)	43.92 ± 11.25	11.93 ± 13.22	−0.065	.031^∗^
Treg cell (%)	1.59 ± 2.27	−0.19 ± 1.13	−1.604	.109

### 3.4 Correlation of differences of white blood cell count, lymphocyte, and peripheral blood T lymphocytes with severity in HBV-ACLF patients after 4-week integrative treatment

**Figure 4:**
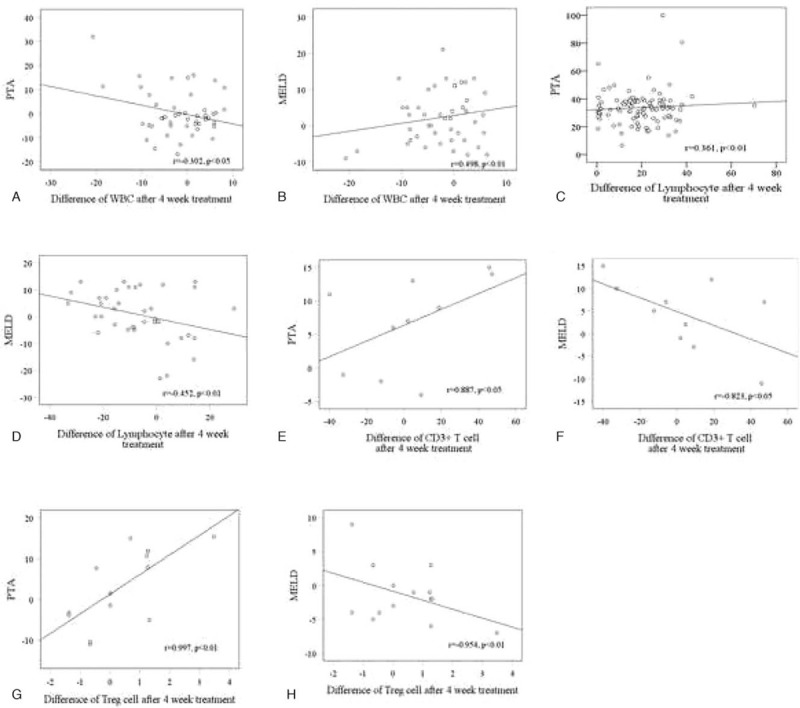
Correlation of white blood cell count, lymphocytes, and peripheral blood T lymphocytes with severity in HBV-ACLF patients after 4-week integrative treatment. Spearman correlation analysis represents the correlation between indicators. A, White blood cell count is negatively correlated with PTA. Statistical significance: *P* < .05. B, White blood cell count is positively correlated with MELD score. Statistical significance: *P* < .01. C, Lymphocytes are positively correlated with PTA. Statistical significance: *P* < .01. D, Lymphocytes are negatively correlated with MELD score. Statistical significance: *P* < .01. E, CD3^+^T cells is positively correlated with PTA. Statistical significance: *P* < .01. F, CD3^+^T cells are negatively correlated with MELD score. Statistical significance: *P* < .01. G, Treg cells are positively correlated with PTA. Statistical significance: *P* < .05. H, Treg cells are negatively correlated with MELD score. Statistical significance: *P* < .05.

Sixty-six patients received 4-week integrative treatment. We also used Spearman rank correlation analysis to examine the associations of white blood cell count, lymphocytes, and peripheral blood T lymphocytes with the severity of HBV-ACLF patients after treatment. Results indicated that differences of white blood cell count after treatment were negatively correlated with PTA (Fig. 4A), while PTA were positively correlated with lymphocytes, CD3^+^ T cells, and CD4^+^CD25^+^ Treg cells (Fig. 4C, E, and G). Differences of white blood cell count after treatment were positively correlated with MELD score (Fig. 4B), while MELD scores were negatively correlated with lymphocytes, CD3^+^ T cells, and CD4^+^ CD25^+^ Treg cells (Fig. 4D, F, and H). The results indicated that the differences of white blood cell count, lymphocytes, and peripheral blood T lymphocytes in HBV-ACLF patients after the treatment were obviously correlated with severity of HBV-ACLF disease, and that the immune function was improved, contributing to the improvement of HBV-ACLF patients.

### 3.5 Response of laboratory indicators to prognosis of HBV-ACLF after 4-week integrative treatment

After the 4-week integrative treatment, this study was divided into effective group and ineffective group according to the guidelines for the diagnosis and treatment of liver failure.^[19]^ The endpoint was judged as the patient's discharge status or death at the hospital. We examined whether the characteristic indicators were associated with prognosis in HBV-ACLF patients. ROC curve was used to analyze the prognostic value. The area under the curve of TBIL, ALT, INR, MELD, and white blood cell count was significantly larger than those under the diagnostic reference line (*P* < .05) (see figure, supplemental Fig. 1A, which demonstrated the altered indicators in patients). As for the area under the curve of Alb, serum sodium, and creatinine, there were no significant differences (*P* > .05) (see figure, supplemental Fig. 1B, which demonstrated the altered indicators in patients). The ROC curve parameters were shown in Table 3. Results suggested that the laboratory indicators such as TBIL, ALT, INR, MELD, and white blood cell count were potential criteria, which could design an accurate prognostic score for patients with HBV-ACLF.

**Table 3:** ROC curve parameters of laboratory indicators in HBV-ACLF patients after 4-week integrative treatment.

Variables	AUROC	*P* value	95% CI
TBIL	0.890 ± 0.052	.000^∗∗∗^	0.787 to 0.992
ALT	0.702 ± 0.083	.029^∗^	0.538 to 0.865
INR	0.781 ± 0.076	.002^∗∗^	0.631 to 0.930
WBC	0.796 ± 0.074	.001^∗∗∗^	0.652 to 0.940
MELD	0.901 ± 0.051	.000^∗∗∗^	0.801 to 1.000
Alb	0.49 ± 0.09	.316	0.41 to 0.78
Creatinine	0.432 ± 0.08	.112	0.56 to 0.90
Serum sodium	0.286 ± 0.08	.121	0.12 to 0.45

## 4 Discussion

Limited retrospective studies and APASL consortia indicated that ACLF in patients with HBV etiology will have a very high mortality due to the liver failure.^[7,20]^ Changes in the immune system in patients with HBV-ACLF are closely related to the responsiveness of patients’ conditions.^[21]^ There is a growing consensus that the host immune response, especially the virus-specific T cell response, is the key determinant influencing the course of disease and the onset of liver disease.^[11]^ Impaired peripheral immune response to microbial challenges is postulated to be responsible for the development of secondary infections, which is an independent predictor of mortality in patients with ACLF.^[22]^

Recent studies have shown that the activation of immune system results in immune cell activation and secretion of large amounts of cytokines, and these cytokines play an important role in the development of liver failure.^[23]^ Previous studies showed that the pathogenesis of the disease is roughly classified into 2 aspects: direct damage and immune mediation,^[24]^ where cellular immune disorders may be the key to the occurrence of liver failure. In cellular immunity, T lymphocytes are the main effectors, which contain different subpopulations to complete the immune response to exert immune regulatory functions.^[25,26]^ The occurrence of liver failure is caused by the killing effect of the cells during the process of clearing virus.^[3,21,27]^ Studies have shown that the cytotoxic T lymphocytes play a significant role in the process of killing virus after HBV infection.^[12]^

In our study, HBV-ACLF patients showed different characteristics with normal people and CHB patients: the total number of peripheral white cells significantly increased, and the proportion of peripheral blood lymphocytes and T lymphocytes significantly decreased, indicating that in patients with HBV-ACLF, their bodies’ immunity is in immune depletion state. Correlation analysis also showed that the characteristics of peripheral white cell count, lymphocytes, and T lymphocytes in patients with HBV-ACLF were related to the pathogenesis of the disease. The above clinical characteristics indicated that the changes of immune system for an appropriate alternative definition of HBV-ACLF could be clinically helpful for early diagnosis.

After enrolling patients with HBV-ACLF having the 4-week integrative treatment, correlation analysis showed that the differences of peripheral white cell count, lymphocytes, and T lymphocytes in patients with HBV-ACLF were related to the pathogenesis of HBV-ACLF. These results suggested that the effects of the integrative treatment on the immune system were related to the pathogenesis of the disease. Changes in white blood cells, lymphocytes, and peripheral T lymphocytes in patients with HBV-ACLF were closely related to prognosis: the increasing trend of total white blood cells and the decreasing trend of lymphocyte and T lymphocyte frequency were slower. The slower the patient's progressed, the better the prognosis. On the contrary, the faster the disease progressed, the more severe the condition and the worse the prognosis. Then, we assessed the prognosis factors through the endpoint of the patients after the treatment. Results showed that the laboratory indicators such as TBIL, ALT, AST, INR, MELD, and white blood cell count were potential criteria for designing an accurate prognostic score for patients with HBV-ACLF. These findings provided additional prognostic information for patients receiving early and intensive management.

Abnormal immune response and immune imbalance is one of the important pathogenesis of ACLF. The study of immune state in patients is of positive significance to the occurrence, development, prognosis, and therapeutic effect of ACLF. At present, the prognosis of ACLF is mainly judged by the prognosis score system composed of various laboratory indexes reflecting liver function. In recent years, new markers reflecting the prognosis of ACLF continue to emerge, which is helpful to improve the existing liver function evaluation system and judge the prognosis. Our results showed peripheral T lymphocytes may contribute to predict the severity of HBV-ACLF, and provide a prognosis response to improve the treatment of HBV-ACLF. But to find out the markers that more accurately reflect the severity of ACLF and related to prognosis, and to improve the prognosis evaluation system still need to confirm and do a lot of research work.

## 5 Conclusion

In summary, our results could help predict risk factors for the severity of HBV-ACLF, and provide a prognosis criterion for patients. It could also provide potential treatment method by immunotherapy targeting peripheral T lymphocytes.

## Acknowledgments

This work was supported by the Leading Talent Project of Jiangsu Province Traditional Chinese Medicine (SLJ0216), the National Natural Science Foundation of China (81870423, 31401210, 31571455, 31600653, and 81600483), the Major Project of the Natural Science Research of Jiangsu Higher Education Institutions (19KJA310005), the Open Project of Jiangsu Key Laboratory for Pharmacology and Safety Evaluation of Chinese Materia Medica (JKLPSE201815 and JKLPSE 201804), and the Project of the Priority Academic Program Development of Jiangsu Higher Education Institutions (PAPD).

## Author contributions

**Author contributions:** Study concept and design: Shanzhong Tan, Shizhong Zheng. Acquisition, analysis and interpretation of data: Feixia Wang, Weiwei Sun, Qian Xiao, Chongfeng Liang, Shulian Jiang, Yanan Lian, Jiangjuan Shao.

**Conceptualization:** Shi zhong Zheng.

**Data curation:** Feixia Wang, Weiwei Sun, Qian Xiao, Chongfeng Liang, Shulian Jiang, Yanan Lian.

**Drafting the manuscript:** Feixia Wang.

**Formal analysis:** Yanan Lian.

**Funding acquisition:** Shi zhong Zheng.

**Guarantor of the article:** Shanzhong Tan, Shizhong Zheng.

**Obtained funding:** Shanzhong Tan, Shizhong Zheng.

**Supervision:** Shanzhong Tan, Shi zhong Zheng.

**Validation:** Jiangjuan Shao, Shi zhong Zheng.

**Visualization:** Feixia Wang.

**Writing – original draft:** Feixia Wang, Shanzhong Tan.

**Writing – review & editing:** Feixia Wang, Shanzhong Tan.

## Supplementary Material

Supplemental Digital Content

## Supplementary Material

Supplemental Digital Content

## Supplementary Material

Supplemental Digital Content
