# Supplementary material for: Peripheral T lymphocytes predict the severity and prognosis in patients with HBV-related acute-on-chronic liver failure
Source: Medicine (Baltimore). 2021 Feb 5;100(5):e24075. doi: 10.1097/MD.0000000000024075 (PMC7870253; doi:10.1097/MD.0000000000024075)
Supplement: Supplemental Digital Content [file medi-100-e24075-s001.docx]

Supplemental Table 1 Baseline demographic and clinical characteristics of enrolled patients with HBV-ACLF (n=97).

| Variables | HBV-ACLF (n=97) |
| --- | --- |
| Age (years) | 40±11 |
| Male, n (%) | 68 (70.10%) |
| HBV-DNA (IU/mL) |  |
| ≤500, n (%) | 13 (13.40%) |
| 500-2×10^6^, n (%) | 84 (86.60%) |
| WBC (10^9/L) | 6.94±3.10 |
| TBIL (μmol/L) | 320.99±151.96 |
| Alb (g/L) | 469.72±615.39 |
| ALT (IU/L) | 164.60±170.45 |
| PTA（%） | 29.41±7.41 |
| Serum sodium (μmol/L) | 136.19±5.11 |
| Creatinine (μmol/L) | 79.16±73.43 |
| Etiology |  |
| HBV exclusively, n (%) | 32 (32.99%) |
| HBV plus cirrhosis, n (%) | 16 (16.49%) |
| lost to follow up, n (%) | 49 (50.52%) |
| MELD | 23.26±6.50 |
