# Supplementary material for: Peripheral T lymphocytes predict the severity and prognosis in patients with HBV-related acute-on-chronic liver failure
Source: Medicine (Baltimore). 2021 Feb 5;100(5):e24075. doi: 10.1097/MD.0000000000024075 (PMC7870253; doi:10.1097/MD.0000000000024075)
Supplement: Supplemental Digital Content [file medi-100-e24075-s002.docx]

Supplemental Table 2 Baseline demographic and clinical characteristics of enrolled patients with HBV (n=20).

| Variables | HBV patients (n=20) |
| --- | --- |
| HBV-DNA (IU/mL) |  |
| ≤500, n (%) | 5 (25%) |
| 500-2×10^6^, n (%) | 12 (60%) |
| >2×10^6^，n (%) | 3 (15%) |
| WBC (10^9/L) | 4.98±0.95 |
| TBIL (μmol/L) | 13.13±4.68 |
| Alb (g/L) | 44.12±3.64 |
| ALT (IU/L) | 164.70±166.08 |
| PTA（%） | 86.87±12.14 |
| Serum sodium (μmol/L) | 141.37±2.02 |
| Creatinine (μmol/L) | 72.77±10.40 |
| MELD | 3.26±1.50 |
