# Supplementary figures and images for: Peripheral T lymphocytes predict the severity and prognosis in patients with HBV-related acute-on-chronic liver failure
Source: Medicine (Baltimore). 2021 Feb 5;100(5):e24075. doi: 10.1097/MD.0000000000024075 (PMC7870253; doi:10.1097/MD.0000000000024075)

(A)

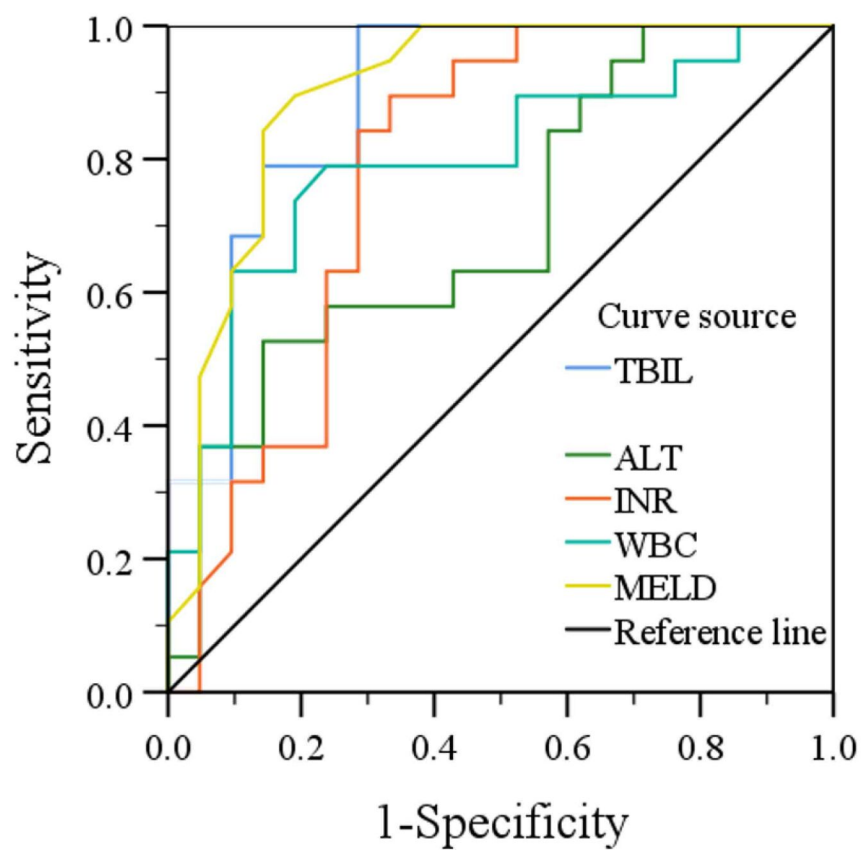

(B)

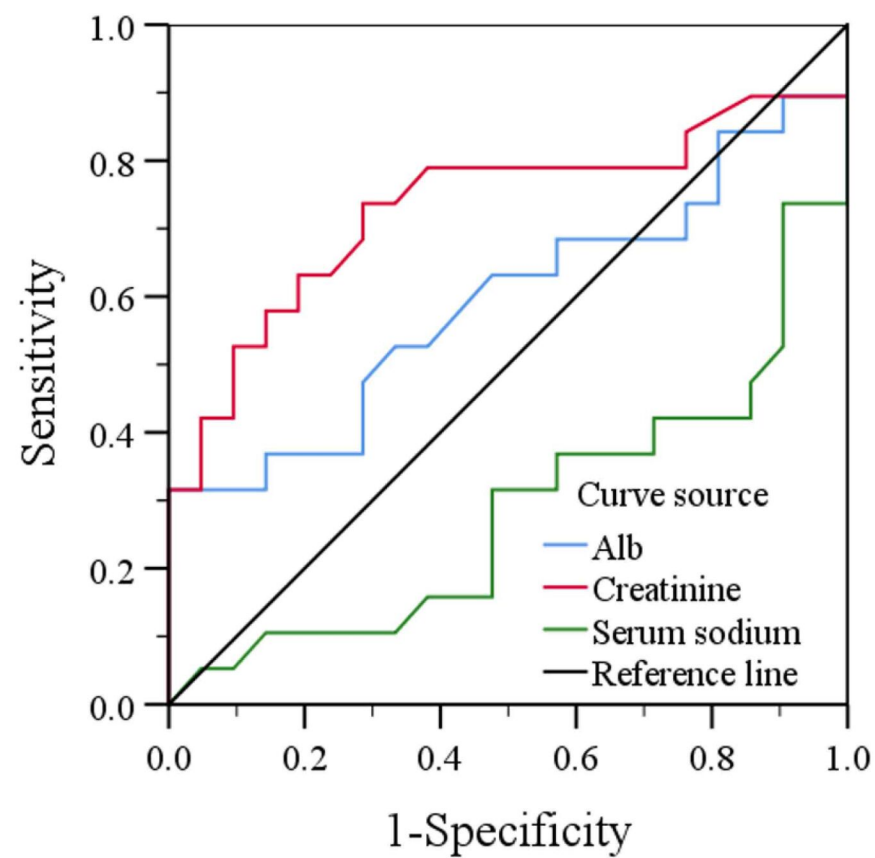

Supplement: Supplemental Digital Content [file medi-100-e24075-s003.pdf]
